# Laboratory capacity for diagnosis of foot-and-mouth disease in Eastern Africa: implications for the progressive control pathway

**DOI:** 10.1186/1746-6148-9-19

**Published:** 2013-01-24

**Authors:** Alice Namatovu, Sabenzia Nabalayo Wekesa, Kirsten Tjørnehøj, Moses Tefula Dhikusooka, Vincent B Muwanika, Hans Redlef Siegsmund, Chrisostom Ayebazibwe

**Affiliations:** 1 National Animal Disease Diagnostics and Epidemiology Centre, Ministry of Agriculture Animal Industry and Fisheries, P. O. Box 513, Entebbe, Uganda; 2 Department of Biotechnical and Diagnostic Sciences, College of Veterinary Medicine, Animal Resources and Biosecurity, Makerere University, P. O. Box 7062, Kampala, Uganda; 3 Department of Environmental Management, College of Agricultural and Environmental Sciences, Makerere University, P. O. Box 7062/7298, Kampala, Uganda; 4 Foot-and-Mouth Disease Laboratory, Ministry of Livestock Development, P.O. Box 18021, Embakasi, Nairobi, Kenya; 5 National Veterinary Institute, Technical University of Denmark, Lindholm, Kalvehave, DK 4771, Denmark; 6 Department of Biology, Ole Maaløes Vej 5, Copenhagen N, DK-2200, Denmark

## Abstract

**Background:**

Accurate diagnosis is pertinent to any disease control programme. If Eastern Africa is to work towards control of foot-and-mouth disease (FMD) using the Progressive Control Pathway for FMD (PCP-FMD) as a tool, then the capacity of national reference laboratories (NRLs) mandated to diagnose FMD should match this task. This study assessed the laboratory capacity of 14 NRLs of the Eastern Africa Region Laboratory Network member countries using a semi-structured questionnaire and retrospective data from the World Reference Laboratory for FMD annual reports and Genbank® through National Centre for Biotechnology Information for the period 2006–2010.

**Results:**

The questionnaire response rate was 13/14 (93%). Twelve out of the 13 countries/regions had experienced at least one outbreak in the relevant five year period. Only two countries (Ethiopia and Kenya) had laboratories at biosecurity level 3 and only three (Ethiopia, Kenya and Sudan) had identified FMD virus serotypes for all reported outbreaks. Based on their own country/region assessment, 12/13 of these countries /regions were below stage 3 of the PCP-FMD. Quarantine (77%) and vaccination (54%) were the major FMD control strategies employed. The majority (12/13) of the NRLs used serological techniques to diagnose FMD, seven used antigen ELISA and three of these (25%) also used molecular techniques which were the tests most frequently requested from collaborating laboratories by the majority (69%) of the NRLs. Only 4/13 (31%) participated in proficiency testing for FMD. Four (31%) laboratories had no quality management systems (QMS) in place and where QMS existed it was still deficient, thus, none of the laboratories had achieved accreditation for FMD diagnosis.

**Conclusions:**

This study indicates that FMD diagnostic capacity in Eastern Africa is still inadequate and largely depends on antigen and antibody ELISAs techniques undertaken by the NRLs. Hence, for the region to progress on the PCP-FMD, there is need to: implement regional control measures, improve the serological diagnostic test performance and laboratory capacity of the NRLs (including training of personnel as well as upgrading of equipment and methods, especially strengthening the molecular diagnostic capacity), and to establish a regional reference laboratory to enforce QMS and characterization of FMD virus containing samples.

## Background

Foot-and-mouth disease (FMD) is a highly contagious, acute, vesicular disease of cloven-hoofed domestic and wild animals [1]. The disease poses significant constraints through reduced productivity and limitation of international trade in live animals and their products [2,3]. The causal agent, foot-and-mouth disease virus (FMDV), belongs to the genus *Aphthovirus,* in the family *Picornaviridae*[4] and exists in seven serotypes; O, A, C, Asia 1, SAT 1, SAT 2 and SAT 3, with all except Asia 1 having occurred in Africa [5,6]. In Eastern Africa, serotypes O, A, SAT 1 and SAT 2 are still in circulation [7-10]. Serotype C was last diagnosed in Kenya in 2004 [11,12] while SAT 3 was last isolated from African buffalos (*Syncerus caffer*) in Uganda in 1997 [13]. However, the FMD situation is constantly evolving necessitating regular typing of currently circulating FMDV strains if effective control measures are to be implemented [14].

The Progressive Control Pathway for FMD (PCP-FMD) tool was developed by FAO/OIE to assist endemic countries to reduce progressively the impact of FMD [15], and consists of six stages (0–5) as shown in Table 1[14]. The main activities of the PCP–FMD tool include: monitoring circulating serotypes, vaccination and enhancing bio-security. In Eastern Africa, quarantine and vaccination are among the existing FMD control strategies [16,17], however, the effectiveness of quarantine is limited by inadequate facilities and very weak law enforcement against animal movements [15,17]. Restriction of animal movements is complicated by social customs (communal grazing, dowry and pastoralism) [17] and both legal and illegal cross-border animal movements. In addition, although, wildlife have been shown to play a role as a maintenance host for FMDV [7], fences and vaccination zones around the national parks are absent. Thus, uncontrolled animal movements are still a major risk for spreading FMD [18] and transboundary mobility of FMDV has been proven between East African countries [9,19]. Hence, there is a need for an integrated regional approach to FMD control [5].

**Table 1:** Description of the PCP-FMD stages

**Stage**	**Description**
0	FMD risk is not controlled/there is no reliable information on FMD
1	Identification of risk and FMD control options
2	Implementation of risk –based control
3	Implementation of control strategy to eliminate circulation (no endemic FMD)
4	Maintenance of zero circulation and incursion with vaccination
5	Maintenance of zero circulation and incursion without vaccination

In the absence of the capacity to control FMD through animal movement restrictions and other biosecurity measures, vaccination remains the only practical control strategy [15]. Vaccination was helpful in the control and eradication of FMD from Europe (up to1991-1992) [20] and, in combination with livestock movement control, helped Namibia and Botswana to obtain FMD free zones without vaccination [5]. However, despite use of vaccination in Eastern Africa in the past few decades, FMD outbreaks are still occurring regularly. The majority of countries in this region use ring vaccination of cattle after confirming an outbreak (protective vaccination) as a control strategy, as opposed to the systematic preventative vaccination schemes recommended for endemic countries. Effectiveness of ring vaccination depends on timely vaccination of all susceptible species [20] and restriction of animal movements which is difficult to accomplish. Moreover, the choice of effective vaccines should be based on matching field strains with available vaccines [21,22], however, due to the lack of the necessary tests for detection and characterisation of field strains [23], such vaccine matching is not commonly done in the region. Instead vaccination against 2–4 serotypes is often carried out in an attempt to ensure protection. In regions where FMD reference laboratories exist, characterisation of field strains is offered in support of regional control or eradication programmes [24,25]. To date, no FMD reference laboratory has been established in the Eastern Africa region to enable systematic characterisation of FMD outbreaks. Consequently, samples from outbreaks in these countries can be submitted to an OIE designated reference laboratory such as the World Reference Laboratory for FMD (WRLFMD), Pirbright, UK, for free typing. However, the extent of regional sample submission and the competences of the existing Eastern Africa NRLs for diagnosis of FMD are not well known.

In this study, we attempt to assess the laboratory capacity of the NRLs for FMD in the countries represented in the Eastern Africa Region Laboratory Network (EARLN) for FMD with regard to sampling, diagnostic tests used, quality assurance and management in pursuit of FMD control.

## Methods

### Study area

**Figure 1:**
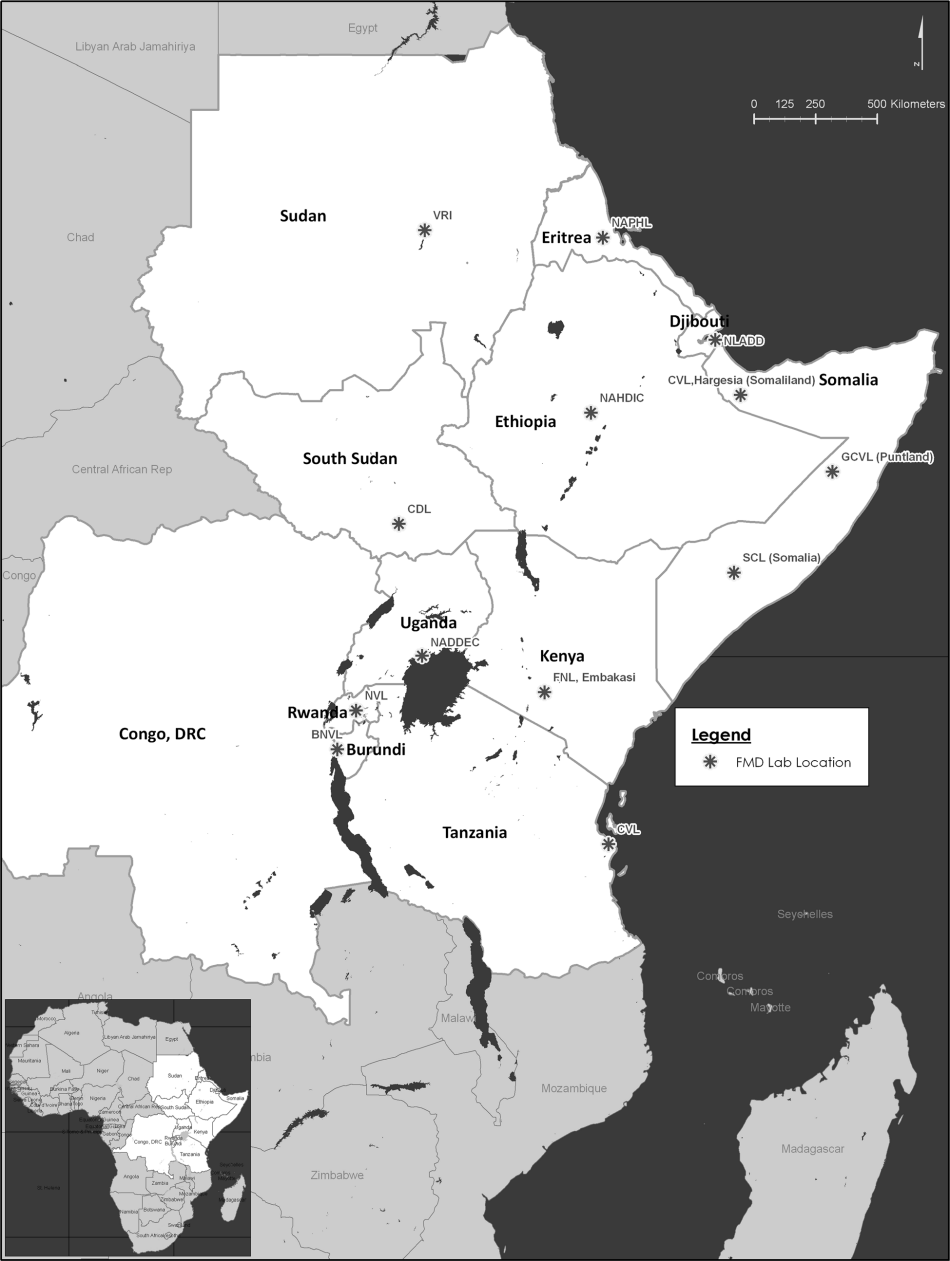
**Study area.** The map shows the location of the 13 participating national reference laboratories (NRL) mandated to diagnose FMD in the Eastern Africa region (members of the Eastern Africa Regional Laboratory Network (EARLN)). The locations were established using Global Position system (GPS) coordinates obtained through the questionnaire. The NRLs include: BNVL: Bujumbura National Veterinary Laboratory in Burundi, NLADD: National Laboratory of Animal Disease Diagnostics, Djibouti, NAPHL: National Animal and Plant Health Laboratory, Eritrea, NAHDIC: National Animal Health Diagnostic and Investigation Center, Ethiopia, FNL: FMD National Laboratory, Embakasi, Kenya, GCVL: Galkayo Central Veterinary Laboratory, Puntland, NVL: National Veterinary Laboratory, Rwanda, SCL: SOWELPA Central Laboratory, Somalia, CVL: Central Veterinary Laboratory, Hargeisa, Somaliland, CDL: Central Diagnostic Laboratory, South Sudan, VRI: Veterinary Research Institute, Sudan, CVL: Central Veterinary Laboratory, Tanzania and NADDEC: National Animal Disease Diagnostic and Epidemiology Centre, Uganda.

The study was carried out among 14 NRLs that handle diagnosis of FMD in 12 Eastern Africa member countries of the EARLN, a network which was established in 2010 mainly to develop the available regional laboratory services and to inform and guide decision makers on control of FMD. Each country had one NRL responsible for FMD diagnosis except for Somalia which encompasses three regions (Puntland, Somalia and Somaliland) with semi-autonomous governments and separate NRLs. So the study endeavoured to cover the 14 NRLs in the region and Figure 1 shows the 13 NRLs that participated in the study which are the: Bujumbura National Veterinary Laboratory (BNVL) in Burundi, National Laboratory of Animal Disease Diagnostics (NLADD) in Djibouti, National Animal and Plant Health Laboratory (NAPHL) in Eritrea, National Animal Health Diagnostic and Investigation Centre (NAHDIC) in Ethiopia, FMD National Laboratory (FNL) in Kenya, Galkayo Central Laboratory (GCL) in Puntland, National Veterinary Laboratory (NVL) in Rwanda, SOWELPA Central Laboratory (SCL) in Somalia, Central Veterinary Laboratory (CVL) Hargeisa in Somaliland, Central Diagnostic Laboratory (CDL) in South Sudan, National Veterinary Research Institute (VRI) in Sudan, Central Veterinary Laboratory (CVL) in Tanzania and National Animal Disease Diagnostic and Epidemiology Centre (NADDEC) in Uganda.

### Study design and data collection

A cross-sectional purposive survey was conducted to assess the laboratory capacity for diagnosis of FMD among the NRLs in Eastern Africa. Data was collected using a semi-structured questionnaire (Additional file 1) sent electronically to one contact person for each of the 14 NRLs through the coordinator of EARLN. The general introduction to the questionnaire and any necessary clarifications were made electronically. The respondents were requested to give information related to FMD outbreaks and control strategies in their countries including: occurrence of FMD outbreaks within the time period (2006–2010), reporting of FMD outbreaks and means of communication, response time for sampling and the personnel involved in sampling, samples collected, time lag in transportation and storage of samples, stage of country on PCP-FMD, FMD control strategies, type and source of vaccines and policies for FMD control. Further, information on tests performed, available equipment for FMD diagnosis, collaborating laboratories and tests requested by NRLs, average number of samples collected annually, cost of laboratory confirmation, biosafety level (BSL), availability of QMS, accreditation status, participation in FMD proficiency testing and inter-laboratory testing within the region, servicing and calibration of equipment, monitoring of sample storage equipment, and staffing and staff development strategies was obtained.

The respondents answered by checking boxes with pre-written options, while additional information could be given in provided spaces. The filled questionnaires were returned electronically through the Coordinator of EARLN and data was entered and analysed using Microsoft Excel®. Furthermore, retrospective data on sample submission and circulating FMDV serotypes were obtained from publically available annual reports of WRLFMD, Pirbright [26] and from GenBank®, National Centre for Biotechnology Information ( NCBI) [27].

This research is part of a larger on -going strategic project ‘Transboundary Animal Diseases in East Africa’. Ethical approval was granted by the Ministry of Agriculture Animal Industry and Fisheries (Reference LHE 199/01), Uganda.

## Results

The questionnaire response rate was 13 out of the 14 contact persons (93%) of the NRLs responsible for diagnosis of FMD in Eastern Africa.

### Annual occurrence of FMD in Eastern Africa (2006–2010)

All countries/regions except Djibouti (12/13) had experienced at least one outbreak in the last five years and seven of these had had outbreaks in each of the 5 years. FMD outbreaks were exclusively reported by the regional zonal laboratories in Ethiopia and by Veterinary officers in three countries (Uganda, Sudan and South Sudan), while for other countries/regions (9/13) both farmers and Veterinary officers were the sources of information about FMD outbreaks to the NRLs.

Table 2 summarizes the number of years with FMD outbreaks, sample submissions to WRLFMD, and the circulating FMDV serotypes in the different countries/regions during 2006–2010 based on data from WRLFMD annual reports and NCBI’s GenBank®. Seven out of 12 (58%) countries/regions that had experienced FMD outbreaks had inconsistently submitted samples to WRLFMD for typing, while only three countries (Ethiopia, Kenya and Sudan) had identified the causal FMDV serotypes for the all outbreaks identified during these 5 years.

**Table 2:** Occurrence of FMD outbreaks in Eastern Africa (2006–2010)

**Country/ Region**	**Number of years with FMD outbreaks**	**Sample submission to WRL (no. of years)**	**FMDV serotypes identified**				
			**2006**	**2007**	**2008**	**2009**	**2010**
Burundi	5	0	-	-	-	-	-
Djibouti	0	0	-	-	-	-	-
Eritrea	5	0	-	-	-	-	-
Ethiopia	5	4	O^ab^	O^a^, A^a^ SAT1^a^, SAT2^a^	O^b^, A^b^	O^b^, A^b^, SAT2^b^	O^b^, SAT2^b^
Kenya	5	4	A^b^, SAT1^ab^ SAT 2^a^	O^a^, SAT2^a^	O^ab^, A^b^, SAT 2^b^	O^b^, A^b^, SAT1^b^, SAT 2^b^	O^b^, A^b^ SAT1^b^, SAT 2^b^
Puntland	nr	0	-	-	-	-	-
Rwanda	1	1	neg^b^	-	-	-	-
Somalia	nr	1	-	-	-	neg^b^	-
Somaliland	nr	0	-	-	-	-	-
South Sudan*	5	0	-	-	-	-	-
Sudan	3	1	A^a^	SAT 2^ab^	O^a^, SAT 2^a^	-	-
Tanzania	5	2	SAT 1^b^	-	-	-	neg^b^
Uganda	5	2	O^a^	O^b^	O^a^	O^a^, neg^b^	-

nr: NRL did not reply to this question, -: no FMDV serotypes identified in a particular year, neg: negative samples, ^a^: data obtained from NCBI, Gene Bank for isolates collected during 2006–2010, ^b^: data from annual reports of WRLFMD, Pirbright for 2006–2010. *: the lack of data from South Sudan reflects the very recent division of Sudan into Sudan and South Sudan in 2011.

### Control of FMD in Eastern Africa

The control strategies for FMD used in these countries/regions are shown in Table 3. Nine countries/regions indicated that they were below PCP-FMD stage 3, while one reported stage 3, two had not yet assessed their stage and one did not indicate its stage of PCP-FMD. With the exception of South Sudan which had no FMD control strategy, two and five countries/regions relied solely on either vaccination or quarantine, respectively, while the remaining five used both vaccination and quarantine. Of the seven countries/regions that used vaccination, only Kenya and Tanzania used pre-outbreak vaccination and post outbreak ring vaccination, while four countries/regions only used post outbreak ring vaccination and one only pre-outbreak vaccination. Only Kenya and Ethiopia had vaccine production plants and with exception of Eritrea, which imported vaccines from Botswana Vaccine Institute (BVI), the other vaccinating countries procured non-purified vaccines from Kenya (data not shown).

**Table 3:** FMD control strategies in Eastern Africa

**Country/region**	**Self assessed stage on PCP-FMD**	**Existing FMD control strategies**
Burundi	0	quarantine
Djibouti	na	quarantine
Eritrea	0	vaccination^a^
Ethiopia	1	vaccination^b^
Kenya	1	quarantine, vaccination^a,b^
Puntland	2	quarantine, vaccination^b^
Rwanda	3	quarantine, vaccination^b^
Somalia	1	quarantine
Somaliland	1	quarantine
South Sudan	na	none
Sudan	1	quarantine
Tanzania	nr	quarantine, vaccination^a,b^
Uganda	0	quarantine, vaccination^b^

na: not assessed, nr: NRL did not reply to this question, a: pre outbreak (preventive) vaccination, b: post outbreak ring vaccination.

### FMD sampling in Eastern Africa

Sampling for FMD was done at different times depending on the purpose. Table 4 summarises sampling in the different countries/regions following reports of FMD outbreaks. In 12/13 countries/regions sampling was done during the acute phase of outbreaks and six and five of these countries/regions also sampled during the subacute and chronic phases of outbreaks, respectively, while the remaining country only sampled during the subacute phase. However, in some countries sampling was also done prior to vaccination (2), post vaccination (1), for research (3) and for surveillance (1). In nine of the 13 countries/regions, sampling was done within 1–6 days after a report of a new outbreak.

**Table 4:** FMD sampling in Eastern Africa

**Country/ region**	**Reporting time for sampling (days)**	**When sampling is done**	**Samples collected**	**Number of samples Collected annually**	**Duration of transport of samples (days)**
Burundi	1-6	AC	Serum, LEF	101-500	1-2
Djibouti	14	SAC	Serum	<100	3
Eritrea	21-30	AC, CH	Serum, LEF	>1000	1-3
Ethiopia	1-6	AC	Serum, LEF, OP	<100	2-7
Kenya	1-6	AC, post V SAC, CH, Res, pre V,	Serum, LEF, OP	101-500	1-2
Puntland	1-6	AC	Serum, LEF, OS	>1000	2-3
Rwanda	1-6	AC	Serum	501-1000	1
Somalia	1-6	AC, SAC	Serum, LEF	>1000	3-4
Somaliland	1-6	AC, SAC	Serum, LEF	>1000	3-4
South Sudan	1-6	AC, SAC	Serum	<100	1-2
Sudan	7	AC, SAC, CH	Serum, LEF, OP	nr	1-3
Tanzania	2-14	AC, SAC, CH, Res	Serum, LEF,OP, OS, WB	101-500	1-2
Uganda	1-6	AC, CH, pre V Res, Sur	Serum, LEF, OP, OS, SA	101-500	1-2

AC: acute phase of outbreak, SAC: sub acute phase of outbreak, CH: chronic phase of outbreak, pre V: pre - outbreak vaccination, post V: post outbreak vaccination, Res: research, Sur: surveillance, LEF: lesion epithelium/fluid, OP: oropharyngeal fluids, OS: oral swabs, SA: saliva, WB: whole blood, nr: NRL did not reply to this question.

Ethiopia exclusively used technicians for sampling, while in Djibouti, Puntland, Somalia, Somaliland and South Sudan field veterinarians participated in the sampling together with the technicians. In other countries (7), officials from NRLs were involved in sampling, either exclusively (Rwanda and Sudan) or together with various combinations of field veterinarians, technicians, animal husbandry officers and officers from zonal veterinary investigation centres/researchers (Burundi, Eritrea, Kenya, Tanzania and Uganda). With the exception of Puntland, all people involved in sampling had been trained in FMD sampling. Serum was collected in all countries, either exclusively (3) or in addition to a combination of lesion epithelium/vesicular fluids, oropharyngeal fluids, oral swabs, saliva and whole blood (10). Of the 12 respondents that answered the question on the annual number of samples, three, four, one and four NRLs indicated that <100, 101–500, 501–1000 and >1000 samples were collected, respectively (Table 4). It took 1–7 days for samples to get to the NRLs and all the 11 respondents who answered the question on sample storage during transit, indicated that samples were kept in cool boxes on ice packs, while three also used liquid nitrogen tanks for storage of virus containing samples.

### Diagnosis and confirmation of FMD in the NRLs

All 13 NRLs were able to diagnose FMD but the reasons for diagnosis of FMD varied: surveillance (12), serotype/vaccine matching (10), confirmation of outbreaks (7), monitoring of vaccine efficacy (2) and research (5) (Table 5). Confirmation of FMD by NRLs was considered a public good in all countries except Burundi, South Sudan and Tanzania (data not shown). The estimated cost for diagnosis of FMD was less than US$50 per sample in all but Eritrea and Rwanda which estimated the cost at more than US$100 (data not shown). The cost for FMD diagnosis was paid by the budgets of NRLs (4), NGO/projects (3) and the ministry either exclusively (3) or in combination with NRLs (1) or projects (2), while in the three countries where FMD diagnosis was not considered a public good, the cost of the services was charged to the submitting body (data not shown).

**Table 5:** Reasons for diagnosis of FMD and tests performed at the NRLs and at the collaborating laboratories

**Country/ region**	**Reasons for diagnosis of FMD**	**Tests performed at NRLs**		**Collaborating laboratories**	**Test performed through Collaboration**	
		**Serology**	**FMDV identification**		**Serology**	**FMDV identification**
Burundi	Cob	ELISA	Ag ELISA	nc		
Djibouti	Sur, Mve	NSP	-	LQCD, Djibouti	NSP LPBE	PCR^a^
Eritrea	Sur	SPCE, LPBE, NSP	PCR^b^, VI	IAH,Pirbright, UK	-	Ag ELISA, PCR ^a^, VI, sequencing
Ethiopia	Sur, Vmat, Res	LPBE, NSP	Ag ELISA, PCR^a,b^	IAH, Pirbright, UK	-	PCR ^a,b^, VI, Sequencing
Kenya	Sur, Vmat, Cob, Mve, Res	VNT, LPBE, NSP	Ag ELISA, CFT, PCR^b^*, VI	IAH Pirbright, UK	-	Ag ELISA, PCR ^a,b^, Sequencing
Puntland	Sur, Vmat,	VNT	Ag ELISA	FNL, Kenya	VNT	-
Rwanda	Sur, Vmat, Cob	LPBE	-	OVI, South Africa Pirbright, UK	LPBE	Ag ELISA, PCR ^a,b^
Somalia	Sur, Vmat	NSP	-	FNL, Kenya	-	Ag ELISA
Somaliland	Sur, Vmat	NSP	-	FNL, Kenya	-	Ag ELISA
South Sudan	Sur, Vmat, Cob	-	Ag ELISA	FNL, Kenya	-	PCR ^a,b**^ , Sequencing^**^
Sudan	Sur, Vmat, Cob, Res	VNT, LPBE, NSP	Ag ELISA, VI	IAH, Pirbright, UK	VNT, LPBE, SPCE	Ag ELISA, PCR ^a,b^, VI, Sequencing
Tanzania	Sur,Vmat, Cob, Res	SPCE, LPBE, NSP	Ag ELISA, PCR ^a,b^*	BVI, Botswana IAH, Pirbright, UK	NSP	Ag ELISA, PCR ^a,b^, VI Sequencing
Uganda	Sur, Vmat, Cob, Res	SPBE, LPBE, NSP	PCR^a,b^	Lindholm, Denmark IAH, Pirbright, UK OVI, South Africa	VNT	Ag ELISA, sequencing, VI

Cob: confirmation of outbreaks, Sur: surveillance, Mve: monitoring vaccine efficacy, Vmat: vaccine matching, Res: research, PCR: Polymerase Chain Reaction, ^a^: real time PCR, ^b^: conventional PCR, VI: virus isolation, Ag: antigen, CFT: complement fixation test, LPBE: liquid phase blocking ELISA, NSP: non-structural protein based antibody ELISA, SPBE: solid phase blocking ELISA, SPCE: solid phase competition ELISA, VNT: virus neutralization test, *:not used for diagnostic but undergoing validation, -: not done, **:Tests performed by IAH, Pirbright, UK through the collaborating laboratory, nc: no collaboration, LQCD: Lab quarantin center-Dammerjog.

Table 5 summarises the diagnostic tests performed by the NRLs. All but CDL of South Sudan used serological diagnostic tests. Only three NRLs (Eritrea, Kenya and Sudan) used virus isolation, eight used immunological detection methods including CDL of South Sudan which exclusively used antigen ELISA and three used nucleic acid recognition method (PCR). Table 5 also shows the collaborating laboratories and tests they perform on behalf of the NRLs. All except BNVL of Burundi had collaborations with other laboratories, and the highest level of collaboration (58%) was registered with the Institute for Animal Health (IAH), Pirbright. The tests provided by the collaborating laboratories were mostly nucleic acid recognition methods (9), while five, eight and six NRLs also requested for virus isolation, Antigen ELISA and serological tests, respectively.

### Quality assurance and standardization for FMD in the NRLs

#### Quality management systems (QMS), biosafety and biosecurity in the NRLs

Eight of the 12 NRLs (67%) that responded to the question on existence of QMS, indicated they existed, while four did not have QMS in place (Table 6). None of the NRLs had been accredited for FMD diagnosis but all except BNVL in Burundi had standard operating procedures (SOP) for FMD diagnosis. Only four of 13 NRLs (31%) participated in annual proficiency tests, while four other NRLs had participated in inter-laboratory testing within the region (Table 6). With regard to biosafety level, most NRLs worked at BSL 1–2, while two (FNL in Kenya and NAHDIC in Ethiopia) were at BSL 3 (Table 6). Five of the NRLs (FNL in Kenya, NAHDIC in Ethiopia, CDL in South Sudan, GCVL in Puntland and NAPHL in Eritrea) had biosafety /biosecurity manuals in place (data not shown).

**Table 6:** Quality management systems (Biosafety levels and quality assessment, equipment maintenance and staffing) among Eastern Africa NRLs

**Country/ Region**	**Bio safety Level (BSL)**	**Quality assessment**			**Equipment maintenance**			**Staff present**		
		**QMS**	**Proficiency testing**	**Inter laboratory testing**	**Regular servi- cing**	**Calibration**	**Monitoring of sample storage daily**	**Veterinarian/scientific supervisor**	**Technical supervisors (manager)**	**Technicians**
Burundi	1	nr	-	**+**	-	-	-	+	-	+
Djibouti	2	-	-	-	+	+	-	+	-	+
Eritrea	2	+	-	-	-	**-**	**+**	**+**	**+**	**+**
Ethiopia	3	+	+	-	+	**+**	**+**	**+**	**+**	**+**
Kenya	3	+	+	-	+	**-**	**+**	**+**	**+**	**+**
Puntland	good	-	-	**+**	+	**+**	**+**	**-**	**+**	**-**
Rwanda	2	-	-	-	-	**-**	**+**	**+**	**+**	**+**
Somalia	low	+	-	**+**	+	**+**	**-**	**-**	**-**	**+**
Somali land	good	+	-	**+**	+	**+**	**-**	**-**	**-**	**+**
South Sudan	1	-	+	-	+	**-**	**+**	**+**	**+**	**-**
Sudan	2	+	-	-	+	**+**	**-**	**+**	**+**	**+**
Tanzania	2	+	-	-	-	**-**	**-**	**+**	**-**	**+**
Uganda	2	+	+	-	-	**-**	**+**	**+**	**-**	**+**

**+**: yes, -: no, nr: NRL did not reply to this question.

#### Equipment servicing and maintenance

Five out of the 13 NRLs (38%) did not regularly service the equipment used in the diagnostic tests for FMD (Table 6), while it was serviced once a year by seven NRLs and twice a year by one NRL (CDL, South Sudan). Six NRLs (46%) calibrated their laboratory equipment yearly and seven monitored their sample storage equipment daily (Table 6).

#### Staffing and staff development

Apart from FNL in Kenya, all NRLs reported a problem of understaffing and one (BNVL in Burundi) had no staff development plans. Ten NRLs had veterinary/scientific supervisors (Table 6) with qualifications ranging from bachelor degree to PhD among their available staff, while three (SOWELPA in Somalia, GCVL in Puntland and CVL in Hargeisa, Somaliland) did not have any University graduates. Seven NRLs had technical supervisors/laboratory managers while six NRLs did not have this middle management level. In five of the seven NRLs with technical supervisors (NAPHIL in Eritrea, FNL in Kenya, GCVL in Puntland, CDL in South Sudan, VRI in Sudan) some of these had only diplomas and certificate qualifications. All NRLs, except CDL of South Sudan and GCVL of Puntland, had technologist/technicians, at four of these including some with only certificate qualification (VRI in Sudan, SOWELPA in Somalia, CVL in Hargeisa, Somaliland and NLADD in Djibouti). Eight NRLs had laboratory assistants of certificate qualification, and six had administrative assistants/support staff (data not shown).

## Discussion

This study showed that FMD is still endemic in the majority of the countries/regions in the Eastern Africa region, and that these countries/regions mainly use quarantine and post outbreak ring vaccination as FMD control strategies. Moreover, the majority of the countries in the region estimated that they were below stage 3 on the PCP-FMD, an important tool for endemic countries to progressively reduce the presence of FMD [14,15].

It has previously been established that disease recognition is essential for any disease control programme [28], and this is particularly relevant for FMD due to the seven FMDV serotypes causing clinically indistinguishable disease [5] and to FMD being easily confused with other viral diseases [29,30]. The present study showed that, in Eastern Africa, the laboratory capacity for FMD, in terms of tests, equipment and skilled manpower, is still limited, and thus all reported outbreaks are not properly serotyped and characterised leading to insufficient knowledge of the regional FMD status. Other factors contributing to unclear FMD status include unwillingness of farmers to pay for diagnosis where it is not a public good (3), and failure (6) or inconsistency (7) in submitting samples to WRLFMD, Pirbright, UK, for free typing, possibly due to logistically complicated and expensive sample shipment [5]. Moreover, three countries had received negative results from WRLFMD, probably due to poor sample quality. Possible reasons for this could be improper handling during the 1–7 day transit from the field to the NRLs, which in 10 of the 13 countries/regions happened in cool boxes with ice packs rather than in liquid nitrogen, or poor quality sample storage in the laboratories caused by unreliable power supplies in most countries/regions. Poor sampling is less likely, since training in sampling technique had been provided in 12 countries/regions, including the three receiving negative results from WRLFMD.

Though three countries in this survey exclusively collected serum during the acute and subacute phases of outbreaks, the majority of the countries/regions followed the recommendation of the OIE [22] and collected samples for demonstration of FMD viral antigen or nucleic acid during the acute phase of outbreaks; moreover, their NRLs had either antigen ELISA or PCR set up. However, success of these tests entirely depends on sample quality determined by timing and handling of samples [1], and a number of outbreaks in the region were not serotyped and/or characterised. All but one of the 13 NRLs relied on antibody ELISAs, most likely because these tests are cheap and suitable for working on many samples, require lower level of biocontainment [22], and neither depend on cell cultures nor on highly sensitive, expensive and service-requiring PCR-equipment [31].

The most widely used antibody ELISAs were tests for identification of antibodies against FMDV non structural proteins (NSP), probably because they are simple and serotype–independent, and thus good screening tests for exposure to FMDV antigen [20,32]. These tests can also differentiate infected from vaccinated animals (DIVA test) [33], however, in Eastern Africa, interpretation of NSP-test results is complicated by the frequent use of non-purified vaccines which elicit antibodies against NSPs, thereby limiting the DIVA application of these tests [20,34,35]. Furthermore, antibodies against NSPs do not appear until day 8–9 after infection [36], thus, to be useful, these tests should only be used for sera sampled in the late subacute and chronic phases. Moreover, as demonstrated in both small ruminants [37] and cattle [33], antibodies against NSPs persist for a long time, and thus NSP ELISAs do not differentiate well between present and past infection at individual level.

Eight of the 13 NRLs serotyped antibodies against FMDV using OIE-recommended tests, i.e. VNT (3), LPBE (7) and SPCE (1), and one of these also used a comparable in-house SPBE developed at the National Veterinary Institute, Lindholm, Denmark [38]. The limited use of VNT is most likely due to lack of cell culture facilities, and possibly also to most NRLs working at BSL 2-level. All available serotype-specific antibody tests, including VNT, show cross reactions [39-41], which have grave implications for the control of FMD in the Eastern Africa countries that greatly rely on serology for serotyping of outbreaks. In endemic situations such cross reactions may be more pronounced due to repeated vaccination against and/or infection with one or more FMDV serotypes [42,43]. Moreover, test related cross-reactivity has been demonstrated for SPBEs in samples collected 1–3 weeks after experimental infection of naive calves (unpublished results) and in field sera from unvaccinated small ruminants [44], and for LBPE in sera from bovines [39], hence, there is a need to improve the specificity of the existing serotype-specific antibody ELISAs.

Tests for detection of FMDV can either identify the serotype directly (antigen ELISA and sequencing) or in combination with other techniques (VI and PCR). In this study, nine NRLs had the capacity for the detection of FMDV while the remaining four NRLs relied on sending samples abroad for antigen ELISA (3) or to another national laboratory for PCR (1). The most widespread test was the antigen ELISA (7), which like the serotype-specific antibody ELISAs, shows cross reactions between the FMDV serotypes [45]. The phasing out of the CFT in the region demonstrates a move towards more modern methods, which is also evident from the five NRLs already using or introducing PCR. Only three NRLs used VI despite this being about as sensitive as PCR [31,46], most likely for the same reasons as for not using VNT. Equally few NRLs (3) used real time and/or conventional RT-PCR in routine diagnosis, which accords with findings in another endemic country, Brazil, where limited use was attributed to lack of infrastructure, high cost and anticipated problems of maintaining technically complicated and service-demanding PCR machines [31].

The majority of the NRLs (12) collaborated with foreign laboratories, including WRLFMD in UK, OVI in South Africa and FNL in Kenya, to complement their own diagnostic services. However, Rweyemamu *et al.*[15] maintained that relying on foreign technical assistance to manage disease control programs may not be sustainable in developing countries, and experience from the region confirms this as the number of samples analysed is insufficient to get adequately detailed knowledge of the circulating FMDV strains to implement sufficiently efficient control measures to reduce FMD in the region. Moreover, recent evidence of the transboundary nature of FMD in Eastern Africa [9] points to a need for assuming a regional approach to achieve more efficient control of FMD and progress on the PCP-FMD.

Eight NRLs had a quality management system (QMS) in place and had participated in laboratory comparisons, either inter-laboratory tests or proficiency tests. In Europe, QMSs are considered essential for diagnosis of FMD [47,48] including focus on competent, motivated staff, organisational management, functional equipment, process control and biosafety/biosecurity [49]. In the Eastern Africa region, although only one of the 13 NRLs entirely lacked SOPs, all had deficient QMS as equipment was not regularly serviced in five and not calibrated in six NRLs, and six did not monitor sample storage equipment daily. This can lead to unreliable equipment and inconsistent quality of samples, which may affect the results of the performed tests [48]. Moreover, none of the NRLs had been accredited for FMD diagnosis, including those in countries with vaccine production plants, and most laboratory comparisons were arranged by laboratories outside the region with a more worldwide focus. Thus, QMS efforts could be strengthened substantially by setting up a regional reference laboratory for FMD, which would arrange local comparative inter-laboratory tests with relevance for the region, encourage QMS and promote virus characterisation among the NRLs [25].

Many diagnostic laboratories also have consultative/advisory and disease surveillance roles [28], and its recommended to build a team of national experts for these tasks [15]. However, in this study, 12 NRLs were understaffed, disclosing a clear regional need to address capacity building in terms of laboratory space, equipment and training of professional and technical staff, as is currently being carried out by collaborative projects in Uganda and Kenya (TADEA, DANIDA-funded) and in Tanzania (SADC TADs, Wellcome Trust- funded), and as has been initiated for the entire region by FAO organizing the NRLs into a network (EARLN).

OIE recommends that FMD diagnosis is carried out in OIE class 4 facilities [25,50] and this is generally adhered to in the FMD-free countries. However, most Eastern African NRLs were working below BSL 3 including seven NRLs undertaking virological tests to diagnose FMD. Moreover, the recommendations for developing biosafety manuals and adopting biosafety policies [51], were only implemented at five NRLs. The low level of biosafety and biosecurity measures could result in escape of FMDV as happened in the 2007 UK FMD outbreak [52], and it may be speculated that a (presumably small) proportion of the outbreaks in Eastern Africa may be due to poor laboratory biocontainment.

## Conclusions

The overall status of FMD in the region remains obscure due to insufficient diagnostic capacity, leading to lack of regular typing of outbreak strains. The NRLs largely depend on antigen and antibody ELISAs, supplemented by varying levels of virus identification and characterisation performed by international laboratories; moreover, they do not prioritize QMSs and none of them are accredited for FMD diagnosis. This calls for improvements at the NRLs, including training of personnel and upgrading equipment and diagnostic methods, to ensure accurate, reliable and correctly interpreted results. More reliable results would also provide an enhanced background for more narrow selection of vaccine strains/serotypes, which would reduce costs of FMD vaccination campaigns and could lead to increased numbers of vaccinated animals.

Moreover, a regionally coordinated FMD control strategy should be implemented to ascertain sustainable impact of national efforts to improve according to the PCP-FMD, including the establishment of a regional reference laboratory to oversee QMS and promote the characterization of FMDV.

## Competing interests

The authors declare that they have no competing interests.

## Authors' contributions

AN, SNW & CA conceived and designed the study; AN was responsible for data analysis, manuscript preparation, review, corrections and submission. SNW further participated in data collection and manuscript reviews; MTD, VBM, KT, HRS & CA participated in the supervision of data analysis and manuscript preparation and critical revision. All authors read and approved the final manuscript.

## Supplementary Material

Additional file 1 Questionnaire on Laboratory capacity for Foot and Mouth Disease (FMD) Diagnosis in Eastern Africa. Click here for file
