# Supplementary material for: Laboratory capacity for diagnosis of foot-and-mouth disease in Eastern Africa: implications for the progressive control pathway
Source: BMC Vet Res. 2013 Jan 24;9:19. doi: 10.1186/1746-6148-9-19 (PMC3562503; doi:10.1186/1746-6148-9-19)
Supplement: Additional file 1 — Questionnaire on Laboratory capacity for Foot and Mouth Disease (FMD) Diagnosis in Eastern Africa. [file 1746-6148-9-19-S1.pdf]

QUESTIONNAIRE ON LABORATORY CAPACITY FOR FOOT-AND-MOUTH DISEASE (FMD) DIAGNOSIS IN EASTERN AFRICA

This questionnaire is being sent to FMD Laboratory Directors or contact persons in the Eastern African countries including Kenya, Somalia, Uganda, Burundi, Rwanda, Democratic Republic of Congo, Djibouti, Eritrea, Ethiopia, Sudan, South Sudan and Tanzania for voluntary assessment of the regional diagnostic and control capacity for FMD in line with the pursuit of the Progressive Control Pathway. The information, kindly provided, will be presented at the upcoming FAO/EUFMD funded East African FMD Laboratory Network (EARLN-FMD) 28 – 29th November, 2011, Entebbe, Uganda, and will augment on-going diagnostic studies, research and control efforts for FMD in Eastern Africa.

NAME OF COUNTRY

NAME OF NATIONAL FMD LABORATORY/ INSTITUTION

NAME OF THE RESPONDENT

POSITION IN THE INSTITUTION

LABORATORY ADDRESS (Location, Email, Telephone, Fax, GPS)

SECTION A: FMD OUTBREAKS AND CONTROL STRATEGY

1. Has your country experienced FMD outbreaks in the last five years? ☐Yes ☐ No

   a) If yes, please mention the number of years and the approximate number of outbreaks per year in the positive years?

   b) How did you get the information about these outbreaks?

   ☐ Telephone calls ☐ Email ☐ Post mail ☐ other, specify

c) Who are the senders of the information about the outbreaks to the national authorities? ☐ Farmers ☐ In-charge veterinary officer ☐ District Veterinary officer ☐ others specify)

d) How long does it take your national laboratory/institution to report for sampling following a report of a fresh FMD outbreaks?

☐ 1-6 days ☐ 1 week ☐ 2 weeks ☐ 3 weeks ☐ 4 weeks ☐ over 1 month

e) Who does the sampling for FMD?

☐ Field veterinarians ☐ Animal husbandry officers ☐ Technicians ☐ Officials from FMD national laboratory ☐ others specify

f) Have the people selected in e) above undertaken a sampling training course?

☐ Yes ☐ No

g) When is sampling for FMD done?

☐ acute phase of outbreak ☐ subacute phase of outbreak ☐ chronic phase of outbreak ☐ Pre-vaccination ☐ Post vaccination ☐ Research ☐ others, specify

h) (i) Which samples are collected?

☐ Lesion epithilum/fluids ☐ probangs ☐ oral swabs ☐ blood for serum extraction ☐ others specify

(ii) How are the samples preserved during transportation to the laboratory? (Indicate sample types, preservatives and storage conditions)

(iii) How long does it take for the samples to get to the laboratory?

i) What FMD control strategies are employed in your country?

☐ Post-outbreak massive vaccination ☐ Post-outbreak ring vaccination ☐ Pre-outbreak vaccination ☐ Quarantine ☐ others (specify)

j) (i) Is there a FMD vaccine production plant in your country? ☐ Yes ☐ No

(ii) If No, what is the source of vaccines that are used in your country? (Indicate name of vaccines, manufacturing company and country)

(iii) What is the purification status of those vaccines? ☐ Purified ☐ non purified

k) Is there any existing policy for control of FMD in your country? ☐ Yes ☐ No

m) At what stage of the Progressive Control Pathway (PCP) is your country?

☐ stage 0 ☐ stage 1 ☐ stage 2 ☐ stage 3 ☐ stage 4 ☐ stage 5

☐ I don’t know

SECTION B: LABORATORY DIAGNOSIS OF FMD

1 a) Give reasons for FMD diagnosis in your country

☐ surveillance ☐ serotype/vaccine matching ☐ confirmation of outbreaks

☐ monitoring vaccine efficacy ☐ research ☐ others (name them)

b) Does your laboratory perform diagnosis of FMD? ☐ Yes ☐ No

c) Name other laboratory (ies) that collaborate with yours for analysis of FMD samples

(Indicate the names and their physical address or full addresses)

d) (i) Which tests are often used in your laboratory to diagnose FMD?

1) Serological tests: ☐ NSP ☐ LPBE ☐ SPBE ☐ SPCE ☐ VNT

☐ Others, (specify)

2) Virological methods: ☐ cell culture ☐ antigen detection ELISA ☐ CFT

3) Molecular methods: ☐ Real Time PCR ☐ Conventional PCR ☐ sequencing

☐ others, specify

(ii) If external partners are used, which tests do they perform for you?

1) Serological tests: ☐ NSP ☐ LPBE ☐ SPBE ☐ SPCE ☐ VNT

☐ Others, (specify)

2) Virological methods: ☐ cell culture ☐ antigen detection ELISA ☐ CFT

3) Molecular methods: ☐ Real Time PCR ☐ Conventional PCR ☐ sequencing ☐ others, specify

g) Which key pieces of equipment are available in your FMD laboratory to facilitate the diagnostic tests that are done in your laboratory? (Tick as many as apply)

☐ real time PCR machine ☐ traditional PCR ☐ LMNP machine ☐ Gel equipment ☐ heating block ☐ bench centrifuge ☐ sequencer ☐ELISA washer ☐ ELISA reader ☐ pipettes ☐ fridges ☐ freezer ($^{-}20^{o}$C) ☐ freezer ($^{-}80^{o}$C) ☐ Orbital shaker ☐ Biosafety cabinet ☐ incubator ☐ others (Name them)

e) (i) Is laboratory confirmation of FMD free of charge (public good) for farmers in your country? ☐ Yes ☐ No

(ii) What is the estimated cost of laboratory confirmation of FMD in a sample in your country? ☐ less than 50 US dollars ☐ 51-100 US dollars ☐ over 100 US dollars (state the approximate amount)

(iii) Who /which budget pays for the laboratory confirmation of FMD suspected samples in your country? ☐ Regional ☐ Veterinary laboratory ☐ Ministry ☐ others, specify

f) On average, how many FMD samples are collected annually in your country?

☐ Less than 100 ☐ 101-500 ☐ 600-1000 ☐ over 1000

SECTION C: QUALITY ASSURANCE AND STANDARDIZATION

1 a) What Biosafety Level (BSL) is your national Laboratory?

b) Does the national FMD laboratory operate a Quality Management System?

☐ Yes ☐ No

c) (i) Is your national Laboratory accredited for FMD diagnosis? ☐ Yes ☐ No

(ii) if yes, Indicate the accreditation body, date and accreditation number

d) Are there standard operational procedures (SOP) for FMD diagnosis in place?

☐ Yes ☐ No

e) (i) Has your laboratory ever participated in FMD proficiency testing? ☐ Yes ☐ No

- If yes: - how often do you do this?

  - Indicate the name/panel and source of test

(ii) Have you ever sent FMD suspect samples to another laboratory in East Africa and compared your results? ☐ Yes ☐ No

- If yes, Indicate the laboratory(ies) name and address

h) Do you have regular service of your laboratory equipment? ☐Yes ☐ No

- if yes; How often do you service them?

☐ as recommended by manufacturer ☐ once a year ☐ twice a year ☐ over one Year ☐ rarely (give the approximate time)

i) Do you regularly calibrate all equipment where manufacturer prescribes calibration in your laboratory? ☐ Yes ☐ No. If yes, how often?

j) How often do you monitor fridges, freezers and liquid nitrogen tanks performance (sample and reagent storage equipment)?

☐ Daily ☐ weekly ☐ monthly ☐ fortnight ☐ yearly ☐ others specify

k) (i) What is the total number of personnel at your national FMD laboratory?

(ii) In your opinion, is this number of personnel sufficient? ☐ Yes ☐ No

(iii) Indicate in the table below the desired number of personnel, the number present and their education levels per category.

Category	Desired number	Number present	Level of education				
			PhD	Masters	Bachelors	Diploma	Certificate
Veterinary/Scientific Supervisors							
Technical Supervisors							
Technologists/ Technicians							
Laboratory assistants							
Administration and support staff							

m) What staff development strategies do you have at your laboratory?

Thank you for your participation

Please send the filled forms or requests for clarifications, to Dr. Sabenzia Nabalayo Wekesa (snabalayo@yahoo.com), Co-ordinator, EARLN-FMD, and copy to Dr. Alice Namatovu (alicenmtv207@gmail.com), PhD Fellow, TADEA.
